# A Fulcrum-Assisted Reduction Technique Using a Novel "Seesaw" Plate Mechanism for Enhanced Stability in Unstable Weber B Ankle Fractures

**DOI:** 10.7759/cureus.96761

**Published:** 2025-11-13

**Authors:** Zaid M Al Ani, Khalid Sharif, Sarvpreet Singh

**Affiliations:** 1 Trauma and Orthopaedics, Peterborough City Hospital, Peterborough, GBR; 2 Trauma and Orthopaedics, Diana, Princess of Wales Hospital, Grimsby, GBR; 3 Lower Limb Arthroplasties, North West Anglia NHS Foundation Trust, Huntingdon, GBR

**Keywords:** bimalleolar ankle fracture, foot & ankle biomechanics, locking compression plates, operative skills, quality improvement research

## Abstract

The management of unstable Weber B ankle fractures presents significant challenges, particularly in patients with osteoporotic bone. Conventional fixation techniques often employ a lag screw to achieve compression at the fracture site. However, in osteoporotic bone, this approach may fail to provide adequate compression and may cause complications such as irritation to surrounding tendons or the distal tibiofibular syndesmosis, sometimes necessitating additional surgery for hardware removal. We describe a novel "seesaw" technique that uses a laterally applied, obliquely oriented locking plate to achieve stable fixation. After initial provisional stabilization with a K wire instead of a lag screw, the distal end of the plate is secured with locking screws. A non-locking screw is then inserted just proximal to the fracture site, serving as a fulcrum. By applying posterior pressure to the plate, a "seesaw" motion is induced, translating the distal fibular fragment anteriorly. This maneuver enables compression across the fracture site and restores anatomic alignment. Final fixation is completed with additional locking screws. Over a three-year period, this technique was employed in more than 50 patients with unstable Weber B fractures. No cases of syndesmotic instability, malalignment, or loss of reduction were observed during the rehabilitation period. This method provides a stable, reproducible alternative to standard fixation techniques and is especially advantageous in osteoporotic bone. It allows for precise anatomic reduction, sustained fracture compression, and early mobilization, potentially reducing complication rates and the need for secondary procedures.

## Introduction

Unstable Weber B ankle fractures are commonly associated with disruption of the ankle mortise and syndesmotic instability [1,2]. Achieving and maintaining anatomical reduction in such fractures is critical to restoring joint congruence and preventing post-traumatic arthritis. The traditional method of fixation uses interfragmentary compression with a lag screw, followed by neutralization with a lateral plate. However, this construct can be associated with several limitations.

In osteoporotic bone, lag screw fixation can be technically demanding and may fail to achieve or sustain adequate compression due to poor cortical purchase, increasing the risk of screw cut-out or loss of reduction [3-5]. Moreover, hardware irritation, fracture comminution, and potential syndesmotic disturbance are recognized complications that may necessitate secondary surgery for hardware removal [4,5].

To overcome these challenges, we developed a fulcrum-assisted “seesaw” plate technique that enables controlled fracture compression while maintaining mortise integrity and syndesmotic alignment. This approach eliminates the need for a lag screw to sustain interfragmentary compression and offers enhanced stability, particularly in osteoporotic bone.

This article describes the surgical technique in detail and presents early clinical outcomes in a cohort of patients treated with this method.

## Technical report

**Figure 1:**
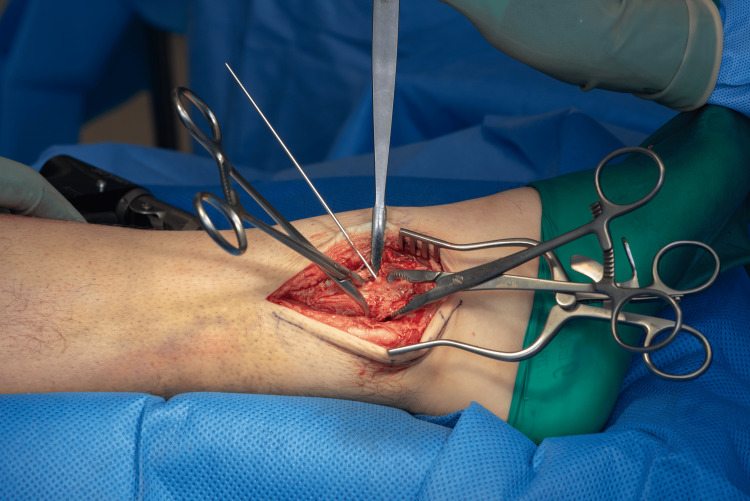
Initial reduction and temporary K wire fixation.

**Figure 2:**
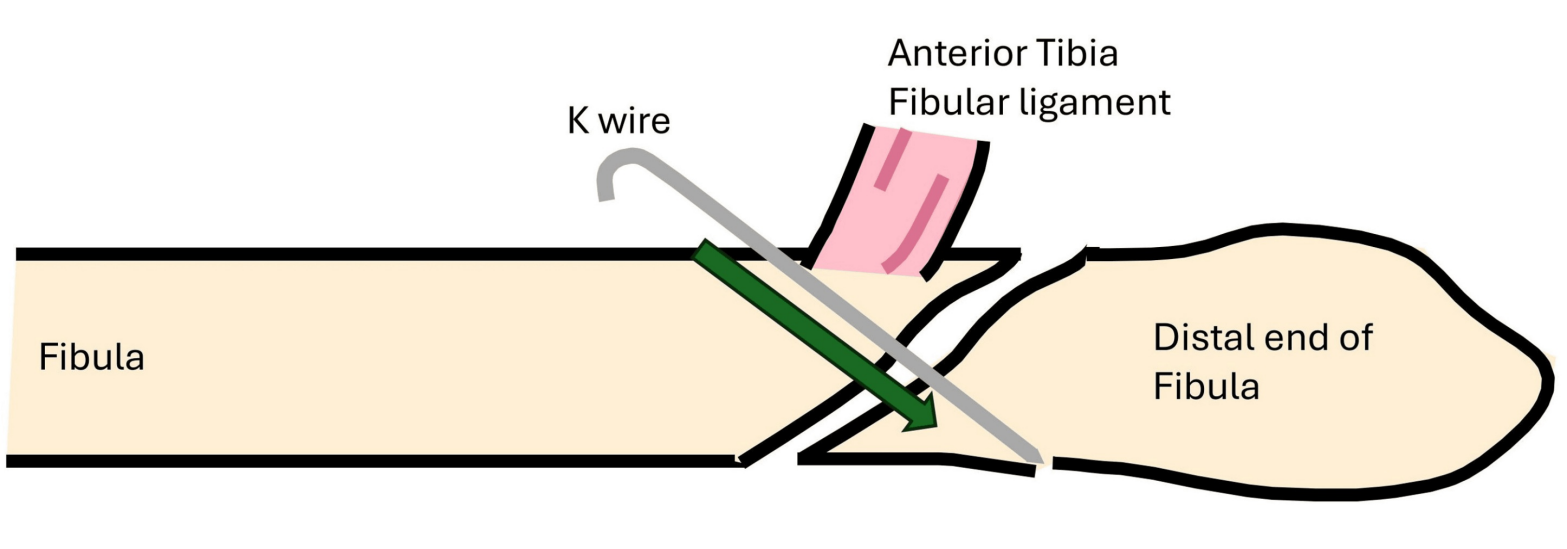
Initial reduction and temporary fixation.

A standard lateral approach to the fibula is performed. Following anatomical reduction, a K-wire was inserted across the fracture to temporarily hold the reduction (Figures 1-2). This step maintains alignment during application of a locking plate, as lag screw/K wire alone do not provide sufficient or sustained compression in osteoporotic bone [5].

**Figure 3:**
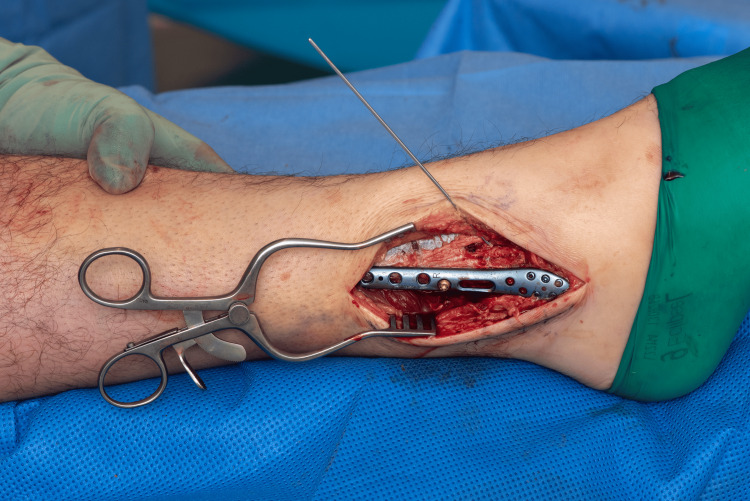
Initial reduction held with a K wire plate in oblique orientation.

**Figure 4:**
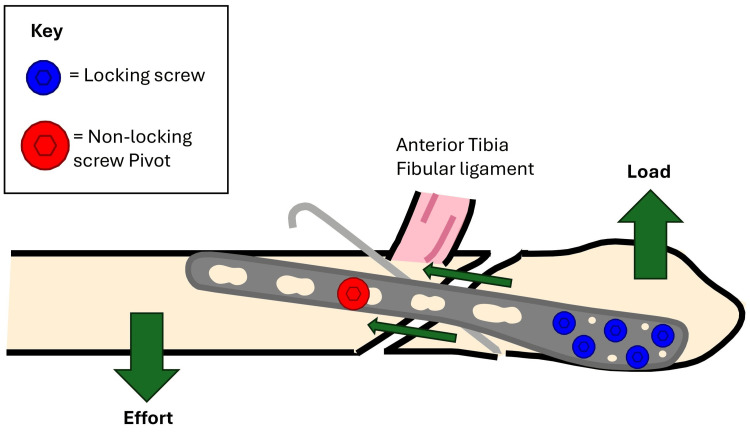
Plate applied in oblique orientation preparing for the "seesaw" maneuver.

An appropriately sized lateral locking plate is selected and applied in an oblique orientation - its distal end positioned posteriorly on the distal fibular fragment, and the proximal end angled anteriorly on the proximal fragment. The distal portion of the plate was secured with locking screws, providing rigid fixation (Figures 3-4).

**Figure 5:**
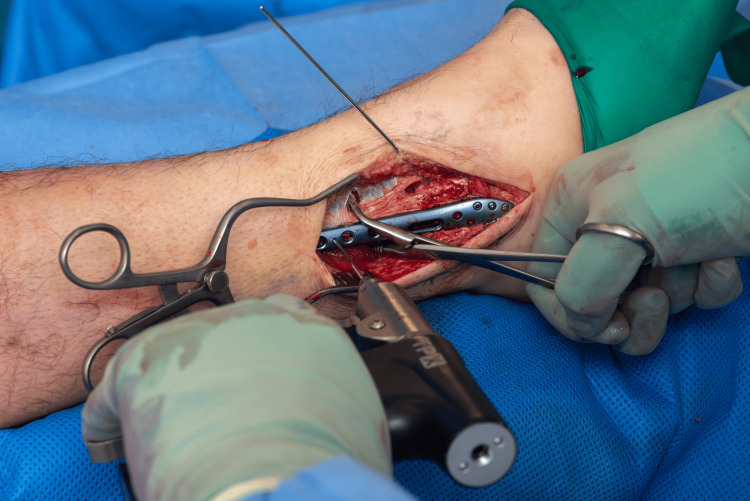
Adding compression to the initial reduction.

A non-locking cortical screw was then inserted into the first proximal hole above the fracture and left partially tightened, serving as a pivot point (Figures 3-4). The proximal end of the plate was pulled posteriorly, using the partially tightened screw as a fulcrum-leveraging the principle of a type I lever (seesaw) [6]. This maneuver translates the distal fibular fragment anteriorly, compressing the fracture site and restoring fibular alignment (Figure 5).

**Figure 6:**
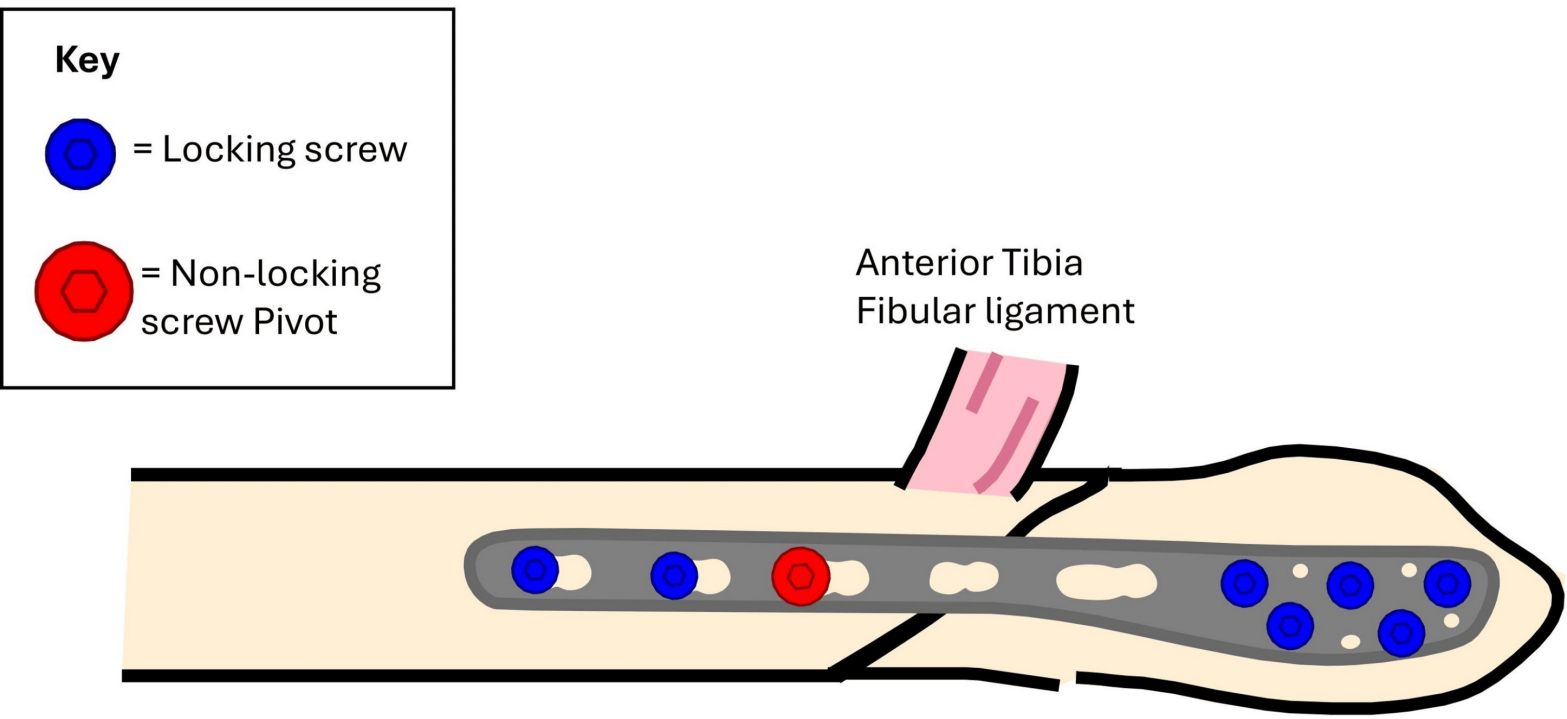
Fracture reduced and compressed following the "seesaw technique".

**Figure 7:**
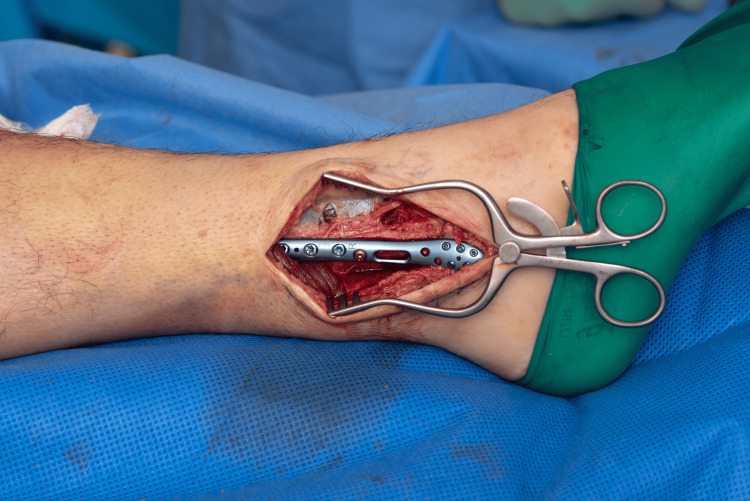
Final fixation after the reduction and compression wire was removed.

Once satisfactory reduction and compression are confirmed, the pivot screw was fully tightened, and at least two additional locking screws were placed proximally to complete fixation. The temporary K-wire or lag screw was then removed (Figures 6-7).

Syndesmotic integrity was assessed intraoperatively using a hook test. In all treated cases, this procedure has provided better stability to the fracture early rehabilitation, with a low complication rate for these patients.

## Discussion

We present a novel technique for managing unstable Weber B ankle fractures in osteoporotic bone. This method introduces an additional function to the lateral locking plate - using it not only for stabilization but also as a fulcrum to generate compression across the fracture, employing a controlled anti-glide or anti-rotation mechanism.

Traditionally, open reduction is followed by interfragmentary compression, typically using a lag screw in combination with a neutralization plate [1,2]. However, this construct is vulnerable to screw pull-out, loss of compression, and subsequent failure of reduction, particularly in osteoporotic bone. Effective lag screw compression depends on solid cortical purchase, which is compromised in osteoporotic patients due to the thin, brittle cortical shell. This leads to a higher risk of screw cut-out, deformation, and, ultimately, loss of fixation [5].

Locking plate constructs, by contrast, do not rely solely on friction between the plate and bone. Each locking screw functions as a fixed-angle device, transforming shear forces into compressive or angularly stable loads while distributing stress across multiple fixation points. This offers a significant biomechanical advantage in osteoporotic bone and provides superior resistance to torsional and shear forces. Previous studies have demonstrated the effectiveness of locking plates in maintaining alignment in such cases, especially when used as neutralization devices [5]. Nonetheless, when reduction is inadequate - whether due to poor bone quality or initial malreduction - mechanical failure and poor outcomes remain a concern [7].

Our “seesaw maneuver” addresses this challenge by using the plate as a mechanical lever to generate controlled, fracture-site compression. This maneuver improves alignment and adds stability without over-reliance on lag screws. It also preserves crucial anteroposterior alignment of the fibula on lateral imaging, which is a key determinant of ankle congruence and has been emphasized in the literature [8].

The lag screw used in Weber B fracture can be a potential for further complications and the need for further surgery for hardware removal; nevertheless, it can be technically demanding, while further comminution during application can compromise stability. Our plate-based seesaw technique offers a “rigid-but-physiologic” alternative.

We still recommend that a K wire can obtain initial interfragmentary alignment or a lag screw temporarily, to provide the space before applying the plate in the described fashion.

Despite its advantages, this technique is not without limitations. Overly rigid constructs can create stress risers, increasing the risk of secondary fractures. Therefore, thoughtful selection of plate design, screw number, and screw placement is essential. In highly comminuted fractures, extremely osteoporotic bone with very thin cortices.

In our clinical series of more than 50 patients treated using this technique over three years, no instances of loss of reduction, malalignment, or syndesmotic instability were observed during follow-up. These early results are encouraging and suggest that the technique is both safe and effective. However, further biomechanical testing and prospective comparative studies are needed to validate its long-term efficacy, reproducibility, and functional outcomes - particularly in elderly and osteoporotic populations.

## Conclusions

The findings from our series indicate that, in Weber B fibular fractures, anatomically contoured locking plate constructs - especially when applied with a fulcrum or anti-glide mechanism - provide superior maintenance of fracture reduction and enhanced resistance to rotational and shear forces compared to conventional lag screw with neutralization plate constructs. This advantage is particularly notable in osteoporotic bone. Additionally, this technique eliminates the need for a lag screw, reducing associated technical challenges and potential complications.
